# American Medical Association

**Published:** 1891-08

**Authors:** 


### AMERICAN MEDICAL ASSOCIATION.

*Tuesday, May 5, 1891.—Morning Session.*

### SECTION OF ORAL AND DENTAL SURGERY.

The section was called to order on Tuesday, May 5, by Dr. E. S. Talbot, of Chicago, Ill., the chairman of the section. Dr. Custer was elected secretary *pro tem.*

*Dr. Talbot.*—It may seem to some of those who are in attendance that the number is rather small; but having been present at all the meetings since the section was organized, ten years ago, with one exception, I can say that it never was intended that there should be a large number at these meetings. It was understood that those who were invited to read papers should be men of unusual scientific abilities; and that the papers should have their effect upon the medical profession by being published. For this reason the society has never catered to a large gathering; but there are quite a number of dentists in the city, and to-morrow we may expect to have a larger meeting. There are no minutes; the section simply keeps a record of the papers that are read at the meeting, and the minutes are not intended to be reported at all.

We will therefore dispense with that matter, and the first order this afternoon will be the chairman's address. I will call Dr. J. L. Williams, of Boston, to the chair.

Dr. Talbot then read his address as president of the section.

(For Dr. Talbot's paper, see page 510.)

There being no remarks upon the address, it was passed.

*Dr. Talbot.*—The next paper upon the programme is by Dr. William Carr. In order to facilitate the reading of papers it has been decided to set apart to-morrow afternoon, from three to half passed five, for the purpose of hearing papers by Dr. George S. Allan, of New York, and Dr. R. R. Andrews, of Cambridge; and we have a paper by Dr. A. H. Thompson, of Topeka, Kan. Dr. Thompson was unable to be present.

A discussion then followed in regard to the propriety of appointing a committee to examine papers. They were finally referred to the appropriate committee.

*Dr. Noble.*—I would like to remark that Dr. Edward Maynard died last evening, and I feel it would be highly proper for you to take, at this very first meeting, some steps in reference to his death, and that proper resolutions should be drafted, and that a delegation be appointed to attend his funeral.

*Dr. Andrews.*—I did not know he was so near death, and think the suggestion a good one.

*Dr. Noble.*—I presume the announcement of his death will be in the evening papers. At the last meeting of the section in Washington I know there were quite a number who took a great deal of pleasure in visiting Dr. Maynard, and I think he came to one of the meetings. He certainly was a man deserving of respect and appreciation. I hope that some action will be taken.

*Dr. Williams.*—I move that a committee of three be appointed to draft suitable resolutions on the death of Dr. Maynard, and that as many of the members of the section as possible attend his funeral.

Motion seconded and passed unanimously.

*Dr. Allan.*—I would like to include Dr. W. H. Atkinson with Dr. Maynard in those resolutions.

Motion seconded and passed.

*Dr. Noble.*—I wish to say that our old friend, Dr. Maynard, came to this city about thirty-five years ago. I came here as a young man and as a student, and I well remember at that time with what veneration we looked up to Dr. Maynard, who was then in the very height and pride of his national reputation. I wish to bear personal testimony to his wonderful skill with his fingers; his manipulative ability, not only in the matter of filling teeth, but his knack in the way of making and using tools and instruments in the laboratory. He was one of the most wonderful men I ever knew with reference to executing what his brain thought out; and we all know what a large-brained man he was, and that for many years he was looked upon as one of the leaders in our profession in all the things that pertain to advancement in the way of mechanical appliances. He was one of the first to thoroughly remove the tooth pulp, and probably with more thoroughness than was done before or for some years afterwards. This was mainly due to the care which he took in preparing the fine nerve-broaches which we are now familiar with. I also feel proud of having his name on my diploma along with that of Drs. Harris and Bond. I think there is only one man now living whose name is on that diploma, and that is Professor Sterner. I feel deep sorrow to hear of the death of Dr. Maynard. Of latter years you have not heard very much of him, and I am sorry to see there has been some language used with reference to him that seems to me uncalled for; that he has not taken an active part in societies, his age preventing. I hope we will always be ready to say, peace to the ashes of Dr. Maynard; for he certainly was deserving of veneration; as an operator he stood pre-eminent in our profession. He was always dignified, and, as we all know, bound up with its best interests.

*Dr. Talbot.*—I will appoint on that committee Dr. Noble, Dr. Allan, and Dr. Williams.

The next paper in order will be that written by Dr. A. H. Thompson, of Topeka, Kan., on "The Teeth of Invertebrate Animals." It will be read by Dr. Marshall.

(For Dr. Thompson's paper, see page 519.)

*Dr. Talbot.*—You have all heard Dr. Thompson's paper read by Dr. Marshall. Are there any remarks?

*Dr. Whitfield.*—There is very little I could say on the subject. Last summer I was very much interested in the study, and I have some teeth at home which I think are nearly three-eighths of an inch long and the arrangement is very beautiful. There is a little skeleton-like arrangement, something like a trap, coming to a point, and the tooth presses down over that point. They all work separately.

*Dr. Rusk.*—A few days ago I saw a specimen which I would like to show here, and will bring here some time during the session. It is a very large tooth that was sent by some chief in the West, and the remarkable part of it is that the enamel is black, or almost black. It is a very immense tooth with very short roots. I think it is a very rare specimen.

*Dr. Talbot.*—As the sessions are to be very short and we have to leave the room at half-past five, and as there are a large number of papers, it would seem to be a good plan to have another one this afternoon, so as not to crowd them in the last session; and I would suggest that Dr. Marshall read his paper.

At the suggestion of Dr. Andrews the reading of Dr. Marshall's paper was postponed to a future meeting.

On motion of Dr. Marshall a committee of three was appointed to nominate officers.

*Dr. Talbot.*—I appoint Dr. Marshall, Dr. Taft, and Dr. Williams.

Adjourned.

*Wednesday, May 6, 1891.—Morning Session.*

The meeting having come to order, Dr. Talbot occupied the chair.

*Dr. Talbot.*—The first order of business this afternoon will be the election of officers. According to the constitution and by-laws, officers are to be elected the second day of each annual meeting. A committee was appointed to attend to the matter, and as this is the time for that order we shall take it up at this hour. Have the nominating committee any report to make?

*Dr. Marshall.*—Mr. President, the committee have not been able to get together yet, but we will make a report after the discussion closes.

*Dr. Talbot.*—If there are no other committees ready to report, the next order of business will be papers from Dr. George S. Allan and Dr. R. Andrews.

(For Dr. Allan's paper, see page 525.)

*Dr. Taft.*—Mr. Chairman, the paper that I have just heard seems to me a very good one. It contains points that ought to be regarded and utilized in the treatment or management of the borders of cavities, and especially proximate cavities, which is one of the most important particulars in the matter of filling. It strikes me that in all instances the operator should be guided by the kind of material or tissue he is working upon, or rather the character of the tissue or material. Some teeth are of such good structure that treatment for the arrest of decay is more easy and simple than in others. The importance of thoroughly studying the character of the tissue upon which the dentist expends most of his operative skill should always be kept in mind, whether strong, firm teeth or those of poor, soft structure. It requires very delicate manipulation to secure the best results. I think this is one of the most important points, and a point that is much overlooked by many operators.

Another point that was emphasized, and justly so, was the perfection of finish which ought to be put on the border of every such cavity. It should be polished or dressed with diamond-dust or anything else that will make a perfect finish. Another point is the overlapping of gold. We all know there is a great deal of faulty work done in this direction; a great deal of gold overlapping that ought to be cut away. I think that there should be no more overlapping than there has been cutting of the enamel or dentine. In no instance should gold be permitted to overlap the normal or uncut surface of the teeth.

Another point is the uniform condensation of gold in the cavity. Now, we may say that it is not of great importance, but it is, for if the gold is imperfectly consolidated there is always room for mischief. It should be a matter of study, then, to secure the most uniform condensation, the most perfect adaptation, the most uniform condensation of the gold throughout the cavity. Great skill is required and delicate and intelligent manipulations to secure good results. As to the subject of welding, skilful manipulation is required; the material used must be in good condition; if gold, it must be properly annealed. Any mistake in annealing is fatal. The gold must be free from foreign substance, and it should be pure, and it must be condensed with proper instruments. I believe that nine-tenths of the instruments used are not as they should be. Those only who have manipulated a great deal can tell you how to do it; that is only attained by experience. It is a process that is difficult to definitely explain. The most direct pressure that can be made on the surface is best; if an oblique blow be given, you will very likely destroy the surface for receiving the gold; the best results are attained by as direct pressure as possible. The paper is full of good suggestions.

*Dr. Talbot.*—Any further remarks? Dr. Allan will close the discussion.

*Dr. Allan.*—I wish there were a little more discussion on this subject. I always like putting in contour fillings. I think there is as much conscience required as skill, and no man can do good work unless he puts his conscience into it. I am very glad to have my old friend Dr. Taft so kindly bring forward so many of the points in my paper, and I appreciate it. I will only draw attention now to one point to see if I cannot bring out some more discussion about it; because it is a matter about which I have thought a great deal and I hope with good result to myself. It refers to the face of the teeth. The question I want discussed—and I have not seen any writing about it to any extent—is this question of whether gold ought to be built up overlapping the cavity, or rather the gold shall bulge over the cavity and be finished off. I take the position and believe that it is one that is substantiated, both in practice and in theory, that the building up of the gold overlapping filling simply lying against the face of the tooth is a greater error. Gold in a plastic condition, if over-hammered, becomes springy and does not take position against the face of the cavity that you can claim with certainty as being a perfect joint. In contour filling you hammer it against the side of the cavity, there is unequal pressure brought in condensing it in place, and the gold loses its soft character and becomes springy; and the more you hammer it the more the springy qualities of the gold are brought out, and it will work away from the side of the cavity and not lie uniformly and flatly against it. That point is one I would like very much to hear some discussion about, as to the advisability of allowing the gold to overlap the edges of the cavity to any extent, and if any, to what extent. I thank you very much for your kindness in hearing my paper and for the favorable reception of it.

(To be continued.)

